# Gene Therapy Strategies to Exploit TRIM Derived Restriction Factors against HIV-1

**DOI:** 10.3390/v6010243

**Published:** 2014-01-14

**Authors:** Emma Chan, Greg J. Towers, Waseem Qasim

**Affiliations:** 1 Centre for Gene Therapy, Institute of Child Health, University College London, London WC1N 1EH, UK; E-Mail: emma.chan@ucl.ac.uk; 2 MRC Centre for Medical Molecular Virology, University College London, London WC1E 6BT, UK; E-Mail: g.towers@ucl.ac.uk

**Keywords:** HIV, gene therapy, restriction factor, TRIM, cyclophilin, TRIMCyp

## Abstract

Restriction factors are a collection of antiviral proteins that form an important aspect of the innate immune system. Their constitutive expression allows immediate response to viral infection, ahead of other innate or adaptive immune responses. We review the molecular mechanism of restriction for four categories of restriction factors; TRIM5, tetherin, APOBEC3G and SAMHD1 and go on to consider how the TRIM5 and TRIMCyp proteins in particular, show promise for exploitation using gene therapy strategies. Such approaches could form an important alternative to current anti-HIV-1 drug regimens, especially if combined with strategies to eradicate HIV reservoirs. Autologous CD4^+^ T cells or their haematopoietic stem cell precursors engineered to express TRIMCyp restriction factors, and provided in a single therapeutic intervention could then be used to restore functional immunity with a pool of cells protected against HIV. We consider the challenges ahead and consider how early clinical phase testing may best be achieved.

## 1. Introduction

Although highly active antiretroviral therapy (HAART) has improved life expectancy of HIV-1 patients, such treatment is far from ideal with notable side effects, problems with compliance and the frequent emergence of viral escape mutants. In addition, HAART must be continued for extended periods in most cases. The need for an effective alternative, alongside advancements in gene therapy, has resulted in a large expansion in the field of research for anti-HIV-1 gene therapy. Two broad gene therapy strategies against HIV are under development and a number have reached clinical testing. Firstly, effector cells can be modified using vectors to target and destroy HIV infected cells. For instance, a chimeric antigen receptor formed of the transmembrane and extracellular domains of CD4 fused to the intracellular ζ subunit of CD3 mediated killing of HIV-1 infected cells by targeting the gp120 envelope (Env) protein and has been evaluated in phase I and II clinical trials [1,2]. Secondly, susceptible cells can be modified to protect them against HIV-1 entry and integration using various mechanisms ranging from zinc finger nucleases targeting the CCR5 virus co-receptor [3,4] to the expression of dominant negative proteins disrupting virus lifecycle [5]. It would be most beneficial to target HIV-1 early in its lifecycle, either at the point of entry or prior to integration, to limit cytotoxic effect, reduce opportunities for mutagenic evolution during reverse transcription and to prevent establishment of latent reservoirs [6]. Preliminary results from clinical trials have provided safety and feasibility data and important information about persistence of engineered cells *in vivo* [7,8,9]. In some cases, there are also promising results relating to the efficacy of the treatments, including a survival advantage of modified cells, reduced viral load and improvements in T cell numbers [5,8,10].

Here we describe how natural anti-HIV restriction factors inhibit HIV and consider how these proteins could be adapted, modified and exploited through gene therapy approaches against the virus. Currently identified restriction factors are grouped as follows; APOBEC proteins, tetherin, SAMHD1 and TRIM5α proteins, and most studies have investigated their activity against retroviruses (Table 1). The most promising for therapeutic approaches appear to be TRIM based factors, and these are considered in detail after discussing alternative factors and their limitations.

**Table 1:** Summary of anti-HIV restriction factors and their characteristics.

Restriction Factor	Mechanism	Viral evasion	Disadvantages	Exploitation
APOBEC	Cytidine deamination of viral genome	Vif mediated proteasomal degradation	APOBEC3G induced mutations may promote evolution of HIV	Modified APOBEC3G restricts HIV in T cells and macrophages [11]
SAMHD1	dNTP triphosphohydrolase activity depletes dNTPs and prevents reverse transcription	HIV-2 Vpx causes proteasomal degradation	Antiviral function limited to quiescent cells	Undefined
Tetherin	Prevents HIV release by anchoring budding virus particles	Lysosomal degradation is promoted by Vpu	Reduces, but does not abolish spread of virus.or establishment of reservoirs	Vpu resistant tetherin in cell lines [12]
TRIM5α/TRIMCyp	Targets virus for proteasomal degradation and disrupts uncoating	HIV-1 accessory proteins are unable to counteract TRIM restriction	Species specific restriction; human TRIM5α does not restrict HIV-1	Chimeric TRIM5α and humanised TRIMCyp restriction demonstrated in humanised mice [13,14]

## 2. Innate Immunity against HIV

Higher organisms have evolved a complex immune system where innate immunity acts rapidly after infection to provide protection against pathogens, allowing time for the adaptive immune system to mount a response. The innate system includes interferon (IFN), pattern recognition receptors (PRR) and secreted soluble molecules such as toll-like receptors or complement. 

One aspect of the innate immune system involves the expression of a variety of antiviral restriction factors. These proteins typically involve low levels of constitutive expression allowing a response immediately upon viral infection, although expression is often upregulated following IFN stimulation after viral infection. Independence from cell signaling or cell-cell interaction results in a rapid, first line of defence against invading viruses. Identified restriction factors differ greatly in structure, but have all been subjected to high levels of survival selection during a process of continual evolution alongside relevant viral pathogens. Targets and mechanisms of viral restriction vary between these factors and are generally independent of other cellular processes, not requiring complex signaling pathways to elicit responses in contrast to many other branches of the immune system [15,16]. The expression of restriction factors is far more widespread than initially thought and a broad range of mammals have now been shown to express these proteins as a defence against many different viruses [17,18,19,20]. Typically HIV-1, HIV-2 and SIV are not significantly inhibited by restriction factors of their natural host species as through necessity these viruses have developed circumventing mechanisms throughout evolution. 

Viral infectivity factor (Vif) is critical for HIV-1 infection in certain cell types, including CD4^+^ T cells [21]. Resistance against Vif-deficient HIV-1 is mediated by the apolipoprotein B mRNA-editing catalytic polypeptide-like 3 (APOBEC3) family [22]. APOBEC3G is an enzyme capable of mutating DNA by cytidine deamination [23] and contains *N*- and *C*-terminal cytidine deaminase domains, which mediate RNA binding and sequence specific cytidine deamination of single stranded DNA (ssDNA). Mutation of APOBEC3G catalytic sites leads to a loss of restriction, demonstrating that it functions, at least in part, by editing the viral genome using this activity [24,25]. APOBEC3G is incorporated into the core of Vif deficient virions through interactions with both Gag, specifically the nucleocapsid [26], and viral RNA [27]. Once the virion infects a new cell, APOBEC3G remains associated with the mature viral proteins and RNA to enable deamination during reverse transcription. APOBEC3G also inhibits Vif deficient HIV-1 in a deaminase independent way as mutants with a nonfunctional cytidine deaminase domain are still capable of causing significant restriction [28]. 

Both mouse and African green monkey (agm) APOBEC3G are resistant to HIV-1 Vif and thus mutant APOBEC3G proteins that are resistant to Vif induced proteasomal degradation could be exploited in anti-viral strategies. This species specificity has been mapped to a single amino acid in APOBEC3G, and mutation of the aspartic acid residue at position 128 in human APOBEC3G for lysine renders it resistant to HIV-1 Vif [29,30]. A variation of this Vif resistant APOBEC3G modification is the fusion of human APOBEC3G to a Vpr derived peptide, efficiently targeting it into assembling virions. Despite this modified APOBEC3G still being susceptible to Vif induced proteasomal degradation, the mutant protein is incorporated into virion particles whereupon infectivity is greatly reduced in target T cells [11,31]. 

However, whilst exploitation of APOBEC3G drew initial interest for gene therapy investigators, further studies revealed its interactions with HIV-1 to be highly complex. There is evidence to suggest that sequence mutations induced by APOBEC may actually be beneficial to the virus by promoting drug resistance and evolution. Thus drug resistance mutations develop in HIV-1 in the presence of APOBEC3G *in vitro* [32]. Furthermore, mutant Vif forms that are less able to inhibit APOBEC3G, may be linked to certain protease and reverse transcriptase mutations that confer additional drug resistance [33].

In 2008, two groups identified tetherin (CD317) as a cell-surface protease sensitive restriction factor [34,35]. Tetherin is a type II membrane glycoprotein that inhibits the release of many enveloped viruses, including HIV-1. It functions as a broad specificity restriction factor as it interacts with the host membrane rather than a viral factor [36]. Originally identified on B cells, tetherin has now been shown to have a broad expression pattern, albeit limited in healthy CD4^+^ T cells, dendritic cells and macrophages [37]. HIV-1 normally expresses the accessory protein Vpu, which can abrogate both tetherin induced accumulation of viral particles at the cell surface and NFκB signaling. Vpu is thought to abrogate tetherin through endosomal trafficking and subsequent lysosomal degradation, which requires the host protein βTrCP [38,39]. In addition, Vpu can abolish tetherin mediated restriction without decreasing cellular levels of the protein.

High levels of vector mediated tetherin may be able to saturate Vpu resulting in HIV-1 restriction [40] or Vpu resistant protein variants capable of inhibiting HIV-1 release [12] could potentially be used in gene therapy approaches. However, at best, tetherin based approaches will reduce spread of HIV-1 in modified cells but will not lead to elimination of the virus. Mutant strains deficient in Vpu are likely to be rapidly selected out by positive selection or there may be the evolution of protective proteins seen in other lentiviruses, such as HIV-2 and SIV [41,42,43]. 

Another factor, Sterile alpha motif and histidine/aspartic acid domain containing protein-1 (SAMHD1) was originally identified as a protein associated with Aicardi-Goutières syndrome, an encephalopathic condition that manifests in early childhood [44]. Subsequently, SAMHD1 was found to be an HIV-1 restriction factor and in dendritic cells, macrophages and quiescent CD4^+^ T cells [45,46,47,48]. 

SAMHD1 functions as a dNTP triphosphohydrolase [49] and has been shown to lower dNTP levels sufficiently to prevent reverse transcription [50]. It consists of a conserved N terminal SAM domain and a catalytic HD domain responsible for dNTP triphosphohydrolase activity and nuclease activity against ssRNA, ssDNA and RNA in RNA/DNA hybrids [49,51,52]. Despite providing protection to myeloid cells and resting CD4^+^ T cells, SAMHD1 is also expressed in other cell types, including actively dividing CD4^+^ T cells, but only exhibits antiviral activity in non-cycling cells due to a difference in phosphorylation status [53,54]. This is likely to limit therapeutic strategies likely to be directly successful in actively dividing T cells.

**Figure 1:**
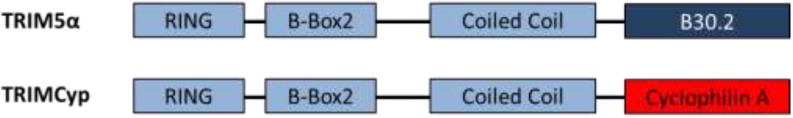
RING, B-Box and coiled coil domains characteristic of TRIM proteins are shown in schematics of TRIM5α and TRIMCyp proteins. In primate TRIMCyp proteins, the B30.2 domain of TRIM5α, which is responsible for capsid recognition, has been replaced with CypA.

In 2004, the cytoplasmic body component TRIM5α was identified as the restriction factor responsible for the resistance of Old World monkeys against HIV-1 [55]. Shortly after this discovery, TRIM5α was confirmed to be responsible for the antiviral activities previously accounted for by Ref1 and Lv1 [56,57], restriction factors identified in humans and primates respectively [58,59,60,61,62,63]. TRIM5α is a member of the large family of TRIM proteins, which has around 100 human proteins with diverse roles. This family is characterised by its TRIpartite Motif (TRIM), which consists of a RING, B-box and coiled coil (RBCC) domains. In addition to the RBCC domains, the longest splice variant, TRIM5α, also includes a C terminal B30.2 or PRYSPRY domain (Figure 1). It is the B30.2 domain that is responsible for binding of restricted virus capsid (CA) and therefore the proteins specificity [64,65,66,67,68]. Typical of antiviral restriction factors, regions within the B30.2 domain have been subject to high levels of positive selection [69] suggesting co-evolution with viruses, with each providing selective pressure on the other to gain the advantage. Throughout evolution it is possible that TRIM5α, with its varying specificity between species, may have played an important role in the control of cross-species transmission of retroviruses [70]. Subsequently, antiretroviral TRIM5α proteins have been identified in other non-primate species, including rabbit [71], cattle [19,20] and hare [72] and restrict various retroviruses in a species specific manner. 

TRIM5 is ubiquitously expressed in all tissues throughout the human body, including T cells [73,74] and while expression levels may be constitutively low, it is upregulated by IFN through a putative IFN-stimulated response element (ISRE) [75]. Restriction patterns are variable between species. For example, TRIM5α from Rhesus macaque (rhTRIM5α) is a strong inhibitor of HIV-1, but not SIVmac, whereas in humans N-tropic murine leukaemia virus (N-MLV) and equine infectious anaemia virus (EIAV) are strongly restricted. Human TRIM5α (huTRIM5α) mediates mild restriction of HIV-2 [76], but does not cause a significant inhibition to HIV-1. These species specific differences in restriction are attributed to CA sequence variation between viruses and subsequently, the ability of TRIM5α to recognise and bind the virus. Alteration of CA amino acid 110 alters specificity of huTRIM5α between B- and N-tropic MLV [77]. There are even different restriction specificities within species, for example in Rhesus macaques there are different TRIM5 alleles which have activity against different retroviruses. Throughout evolution, selective pressure from viral infection has driven diversity in the TRIM5 gene [78,79], although in man, TRIM5α with or without various polymorphisms has little inhibitory effect on HIV-1 infection or disease progression [80,81,82]. Human TRIM5α can be modified to provide specificity against HIV-1. Either substitution with critical rhTRIM5α B30.2 sequences [83] or a single amino acid change at 332 of the human protein is sufficient to activate restriction of HIV-1 [66,67,84]. Although HIV-1 is not susceptible to restriction by native hTRIM5α, unlike the other restriction factors mentioned here, the virus has no accessory protein that is able to counteract the restrictive effects of TRIM5α, making it an ideal candidate for gene therapy approaches against HIV.

**Figure 2:**
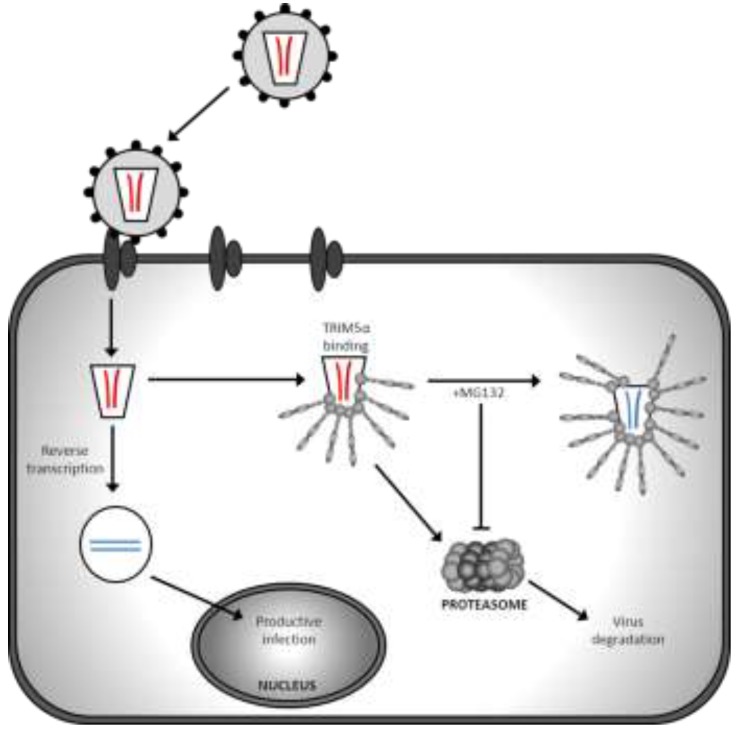
Schematic showing TRIM5α restriction of virus within a cell. TRIM5α is autoubiquitinylated by its RING domain and when virus enters the cell TRIM5α binds viral capsid via its B30.2 domain, targeting the virus for proteasomal degradation. This block to infection occurs prior to reverse transcription. If cells are treated with MG132 to inhibit the proteasome, reverse transcription occurs and the virus is not degraded, but infection is not rescued. This suggests alternative proteasomal independent antiviral mechanisms.

TRIM5α inhibits HIV-1 early in infection, as shown by the absence of reverse transcripts after viral entry [77]. However, the detailed processes by which TRIM5α restricts retroviruses have not been fully determined, although it is likely that TRIM5α mediates restrictive effects through multiple mechanisms (Figure 2). Upon viral entry of a cell, TRIM5α recognises CA via its B30.2 domain, although the interaction between monomers of these two proteins is weak [85]. TRIM5α forms dimers which then spontaneously form hexamers, but this is greatly enhanced in the presence of incoming viral CA. Hexamers are required for efficient restriction and mediate more efficient CA binding with higher avidity by allowing multiple B30.2 domains to cover and interact with the incoming viral core [86]. Binding by TRIM5α in the cytoplasm can lead to accelerated and disrupted uncoating of the virus, preventing infection as this process is normally tightly regulated [87,88,89,90]. Autoubiquitinylation and subsequent proteasomal degradation of TRIM5α requires its RING and B-box domains and it is thought that there is also proteasomal degradation of the TRIM5α-virus complex [91]. Inhibition of the proteasome permits formation of viral reverse transcripts and pre-integration complexes, but these are not detectable in the nucleus. Therefore proteasome inhibition rescues reverse transcription, but does not abrogate the antiviral activity of TRIM5α [91,92,93]. Proteasome independent antiviral pathways are likely to involve sequestering of the viral genome within pre-existing aggregations of TRIM5, or by formation of new aggregates around virus particles. TRIM5α can also be seen to leave cytoplasmic bodies to interact with nearby virus within the cytoplasm [94] and varies between localisation in cytoplasmic bodies and more diffuse distribution throughout the cytoplasm [95]. However, disruption of cytoplasmic bodies with geldanamycin does not prevent restriction, therefore their formation is not essential [96,97]. Recently human and Rhesus TRIM5α have been shown to shuttle between the cytoplasm and nucleus, and inhibition of CRM1 nuclear export machinery results in accumulation of TRIM5α in the nucleus. This is not seen in TRIM5α from cattle, New World monkeys or TRIMCyp [98]. Accumulation of TRIM5α in the nucleus does not abrogate TRIM5 mediated retroviral restriction, although it is likely that residual cytoplasmic protein or newly synthesised TRIM is responsible for restriction. The relevance of nuclear shuttling of TRIM5α is not known, and the protein may have additional undefined functions.

In addition to CA recognition, TRIM5α plays a role in cell signaling in innate immunity. Human TRIM5α and its murine paralog, TRIM30, downregulate NFκB signaling through proteasome independent degradation of TAB2, an adaptor protein upstream of NFκB. In contrast, human and Rhesus TRIM5α are able to activate NFκB expression. The activity of these two opposing effects upon NFκB by the human protein is thought to depend upon TRIM5α levels [99]. For activation of NFκB signaling, TRIM5α functions in conjunction with UBC13-UEV1A, an ubiquitin-conjugating enzyme. Together they assemble unattached K63-linked ubiquitin chains that activate the TAK1 kinase complex. TAK1 subsequently stimulates AP-1 and NFΚB transcription factors involved in innate immune signaling [100]. The formation of ubiquitin chains and activation of NFκB signaling is significantly enhanced upon CA recognition. Prevention of formation of these chains through knockdown of UBC13 or UEV1A abrogates huTRIM5α restriction of susceptible retroviruses, such as EIAV. Through this function, TRIM5α and TRIMCyp act as pattern recognition receptors as recognition of a restricted retroviral CA enhances the activation of NFκB signaling and innate immune responses [100]. 

Another protein that interacts with HIV-1 is the peptidyl-prolyl isomerase (PPIase), cyclophilin A (CypA), which catalyses the switch between *cis* and *trans* conformations of proline residues. CypA interacts with HIV-1 CA at the G89-P90 peptide bond located on a nine amino acid flexible loop (P85-P93), and catalyses isomerisation of the bond [101,102]. This bond is primarily found in the trans conformation (86%) [103].Mutation of either G89 or P90 abrogate CypA binding and in addition, the surrounding residues P85, V86, H87, A88, P93 are also involved in binding [104]. CA interaction results in incorporation of CypA into HIV-1 virions and can be prevented by treatment of producer cells with the immunosuppressive drug cyclosporine A (CsA), which is a competitive inhibitor of CypA, mutation of G89-P90 within the CA or knockdown of the CypA gene. In humans, inhibition of this interaction causes a block to HIV-1 infection soon after cell entry [104,105,106,107], although it is the presence of CypA in target cells, rather than in producer cells, that is required for optimal infection [108,109]. In addition to HIV-1, SIVagm and FIV have also been shown to recruit CypA [110,111,112]. CypA acts upon incoming HIV-1 particles soon after entry and before reverse transcription, at the same time as TRIM5α restriction in non-human primates occurs [113]. CypA has been proposed to have a role in regulating CA uncoating [114]. Also, as CypA causes a range of effects on infectivity, both cell type and species specific, it suggests that other proteins are involved in the interaction between CA and CypA; CypA may provide protection from restriction or make the CA more recognisable to restriction factors such as TRIM5α through isomerisation [115]. In general, disruption of the CypA-CA interaction leads to HIV-1 restriction in human cells but this effect is both strain specific and TRIM5α allele specific [116,117]. Therefore the function of CypA in HIV-1 infection is complicated, even more so because its effect upon lentiviral infection is species specific, as unlike HIV-1 infection of human cells, in many non-human primate cells the CypA-CA interaction actually enhances TRIM5α mediated restriction. CypA mediated CA isomerisation may increase binding affinity of TRIM5α [118,119] or stabilise the CA core prolonging time for recognition by TRIM5α. Most recently, CypA has been shown to have a role in protecting HIV-1 from being detected by the cytoplasmic innate immune DNA sensor cGAS [120]. The HIV-1 CypA binding mutant HIV-1 CA P90A was shown to trigger cGAS in monocyte derived macrophages (MDM) leading to activation of a cell autonomous innate immune response. HIV-1 CA P90A infection of MDM led to production of the second messenger cGAMP, NFkB and IRF3 nuclear localization and production of soluble type 1 IFN, which completely suppressed HIV-1 replication. The importance of IFN in suppressing viral infection was illustrated by rescuing viral replication by blockade of the IFN receptor with specific antibody. The details of how CypA prevents HIV-1 DNA from triggering cGAS remain incompletely described but CypA’s role in recruiting HIV-1 into a specific pathway of nuclear entry involving DNA synthesis in complex with the nuclear pore mediated by interactions between viral proteins and Nup358, TNPO3, CPSF6 and Nup153 is likely to be important [120,121,122].

**Figure 3:**
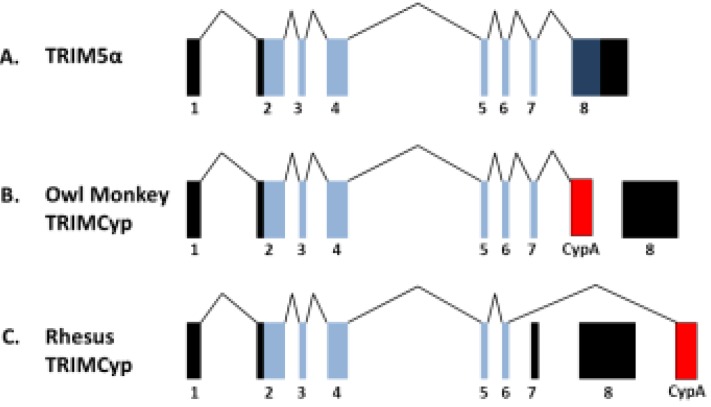
Schematic of TRIM5 and TRIMCyp genes. CypA cDNA retrotransposition into the TRIM5 gene of primates has occurred more than twice throughout evolution to produce in frame fusions of TRIM5 and CypA, with CypA replacing the B30.2 domain. (**A**) Native TRIM5 gene; (**B**) In owl monkeys, CypA insertion in intron 7 produces a TRIMCyp protein consisting of exons 2–7 with CypA replacing exon 8; (**C**) In macaques, CypA is downstream of TRIM5 and accompanied by a mutation leading to skipping of exons 7 and 8. The resultant protein is encoded by exons 2–6 followed by CypA. Untranslated exons shown in black, translated exons in blue, cyclophilin A (CypA) in red.

New World owl monkeys of the genus *Aotus* express an anti-HIV-1 fusion protein comprising the TRIM5 RBCC domain fused to CypA. The fusion has arisen from LINE-1 (L1) mediated retrotransposition of a CypA cDNA into TRIM5 intron 7. This results in an in-frame fusion between exons 2 to 7 of TRIM5 with CypA cDNA, which replaces the B30.2 domain encoded by exon 8 (Figure 1). Owl monkeys are homozygous for this altered gene and do not encode any other TRIM5 alleles. The owl monkey TRIM5-CyclophilinA (omTRIMCyp) fusion protein is a strong inhibitor of HIV-1, due to the ability of CypA to bind HIV-1 CA and recruit the TRIM5 RBCC domains, and is responsible for the resistance of owl monkey cells to this virus. Inhibition can be overcome by treatment with CsA or the use of G89V HIV-1 mutants [123,124]. omTRIMCyp also restricts FIV and SIVagm, but leaves cells from this species susceptible to infection by SIVmac [110]. There has been another incident of retrotransposition of CypA into the TRIM5 locus of Old World macaques species, including rhesus macaques (*Macaca mulatta*), pig-tailed macaques (*Macaca nemestrina*) and crab eating macaques (*Macaca fascicularis*). This second example of a TRIM-Cyp chimera is most likely to have occurred independently of the owl monkey event due to the different location of the CypA coding sequence, with respect to its position in owl monkeys in the TRIM5 gene, downstream of exon 8 in Old World monkeys [125,126,127,128]. The resultant protein is encoded by exons 2 to 6 of TRIM5, with the CypA cDNA replacing exons 7 and 8, in contrast to the owl monkey fusion that is encoded by exons 2 to 7 of TRIM5 and the CypA cDNA (Figure 3). The antiviral specificity of this fusion protein is also distinct to the owl monkey, with the rhesus TRIMCyp being a strong inhibitor of HIV-2, HIV-1 group O and FIV, but not HIV-1 group M. This difference in restriction specificity is due to variations in the CypA domain of rhTRIMCyp compared to that of the genomic CypA, altering the conformation of the active site loop [129]. Furthermore, TRIMCyp alleles from different macaque species also have different antiviral specificities due to additional mutations in their Cyp domains [130]. Further analysis of TRIM5 and CypA genes throughout primate evolution has identified two additional TRIMCyp genes in rhesus macaques, one of which is still expressed in certain Old world monkey species, although as a non-functional protein. These incidences of convergent evolution to produce TRIMCyp restriction factors on more than one occasion and their maintenance in the genome suggests that they experienced strong selective pressure throughout evolution, likely from lentiviral infection [131]. The observation that HIV-1 mutants that do not recruit CypA trigger a cell autonomous innate immune response through activating the DNA sensor cGAS may explain why TRIMCyp chimeras are particularly effective in restricting primate lentiviruses [120].

Despite the differences in specificity of the macaque and owl monkey TRIMCyp proteins, the mechanism of restriction is similar, causing a block in infection before reverse transcription which can be abrogated by CsA treatment or mutation of the TRIMCyp binding site on the viral CA. As with TRIM5α, the mechanism of antiviral restriction is not fully understood, but it is also likely to function through multiple antiviral activities including proteasome recruitment and innate signaling responses. Restriction occurs rapidly after viral entry into the cell before reverse transcription. Although TRIMCyp forms cytoplasmic bodies, they are not essential for restriction [96]. Deletion of the RING domain causes a reduction, but not loss of restriction, suggesting proteasome independent mechanisms, and deletion of the RING and B-Box2 domains eliminates restriction [110] implying that they are important for efficient inhibition. Like TRIM5α, TRIMCyp forms multimers, and dimers, trimers and hexamers have all been identified and TRIMCyp has been shown to disrupt CA cylinders *in vitro*, suggesting antiviral activity through interference with uncoating [89].

## 3. Gene Therapy Exploitation of TRIMCyp Factors

Human mimics of TRIMCyp are able to restrict HIV-1 and confer a survival advantage at levels comparable to the owl monkey fusion protein when expressed in cell lines, primary T cells and macrophages. Delivery of these modified CD4^+^ T cells into an experimental humanised mouse model of HIV-1 showed engraftment of TRIMCyp expressing cells and robust restriction of HIV-1 [13]. Humanised mouse models have also been utilised to investigate human CD34^+^ haematopoietic stem cells (HSCs) transduced with vectors to express chimeric rhesus/human TRIM5α. TRIM5α expressing cells successfully engrafted and gave rise to multilineage progenitors, which were able to protect against challenge with HIV-1 and also showed a survival advantage during infection [132].These chimeric mouse models provide important evidence supporting the use of TRIM5 based gene therapy strategies in clinical trials.

Similar to Neagu *et al*., we have reported similar TRIM5 and CypA fusion constructs and have also described fusions between TRIM21 and CypA, which elicit potent anti-HIV effects [133]. TRIM21 mediates activation of NFκB signaling pathways and the B30.2 domain of TRIM21 is a highly efficient IgG receptor capable of recognising antibody coated virus particles within the cytoplasm and targeting them for proteasomal degradation [134]. After binding to the Fc domain of antibody, the TRIM21 RING domain synthesises K63-linked ubiquitin chains triggering NFκB signaling leading to an antiviral state [135]. These signaling properties are retained in cells transduced to express TRIM21Cyp, suggesting the protein could form the basis of an anti-HIV gene therapy without disrupting endogenous pathways. In addition, TRIMCyp fusion proteins have been generated using alternative TRIM proteins with the same domain structure as TRIM5, including TRIM1, 18 and 19 [136]. Similarly, CypA has been fused to the murine restriction factor Fv1 (Friend virus susceptibility gene 1), retargeting its antiviral specificity from murine leukaemia virus to produce an inhibitor of HIV-1 [137]. A key feature of TRIMCyp based strategies relates to the reduced likelihood of mutagenic escape as the CypA binding loop is highly conserved amongst primate lentiviruses, [110]. Mutation of the CypA binding loop interferes not just with binding of CypA, but also the nuclear pore protein Nup358, which is involved in nuclear import. Disrupting these interactions through mutation of the CypA binding loop affects the site of integration and subsequently viral replication [121]. Therefore, for productive viral infection, maintenance of CypA binding is critical and any escape mutants avoiding TRIMCyp binding are likely to exhibit reduced viral fitness. This appears to be particularly true in macrophages where HIV-1 mutants that do not recruit CypA trigger the DNA sensor cGAS leading to a potent cell autonomous innate immune response that completely suppresses replication [120]. Thus not only will TRIMCyp escape mutants be unfit through an inability to recruit Nup358, they are likely to trigger antiviral and inflammatory cytokine production that will help limit wild type HIV-1 replication in T cells.

Lentiviral delivery of the most promising TRIMCyp offers the prospect of rapid first in man assessment of therapeutic strategies using restriction factors. Phase 1 studies are being planned to investigate the efficiency of gene transfer, engraftment potential and the possibility of host mediated immunes response raised against any novel epitopes formed at the junction between TRIM and CypA. These initial safety studies will transduce and re-infuse autologous T cells and if successful, subsequent therapies will target autologous HSC. Stem cell based approaches will require preparative chemotherapy similar to that used in recent autologous lentiviral mediated gene correction of inherited immune and metabolic conditions [138,139]. Such myeloablation is essential to secure stem cell engraftment and in the context of HIV, may also help eradicate the host lymphoid and myeloid populations that act as a reservoir for HIV. Recent experience from allogeneic stem cell transplantation in patients with HIV-1 supports this premise [140], and whereas transplant based approaches require human leukocyte antigen-matched, CCR5 null donors, an autologous stem cell modification approach will have much wider applicability. Furthermore, if immunity is reconstituted with cells expressing TRIMCyp, both CCR5 tropic and CXCR4 tropic viruses should be restricted. 

For stem cell modification, regulated expression of TRIMCyp would be desirable to avoid toxicity and reduce the risk of insertional mutagenesis. Vectors using internal promoter elements derived from TRIM5 regulatory sequences are under investigation, as are strategies to target TRIMCyp insertion into the endogenous TRIM5 locus. These approaches should result in IFN-inducible TRIMCyp expression in CD4 expressing macrophages and post thymic T cells following exposure to HIV. 

Finally a number of logistical challenges need to be addressed before these approaches can be investigated in man, including the production of clinical grade vector stocks and optimization of *ex vivo* transduction protocols. Nonetheless, there is a compelling rationale to pursue these approaches as supported by encouraging data from other T cell and stem cell gene therapies.

## 4. Conclusions

Despite showing natural ability to restrict certain strains of HIV-1, most identified restriction factors are effectively counteracted by viral accessory proteins. TRIM5α and related TRIMCyp proteins hold the greatest promise for gene therapy applications. Expression of these proteins using lentiviral vectors should protect modified cells through mechanisms that do not appear to be abrogated by any viral countermeasure. 

Early phase clinical trials are planned to investigate the feasibility of TRIMCyp based therapies, and in the first instance will involve *ex vivo* modification and re-infusion of autologous T cells, followed by studies in autologous HSC once feasibility and safety data is established.
